# Nanoscale Mechanisms of Piezoelectric Enhancement in MXene–Fluoropolymer Composites

**DOI:** 10.1021/acs.nanolett.6c02014

**Published:** 2026-07-15

**Authors:** Kalyan Ghosh, Shaashwat Saraff, Sohini Kar-Narayan

**Affiliations:** † Department of Materials Science and Metallurgy, 2152 University of Cambridge, 27 Charles Babbage Road, Cambridge CB3 0FS, United Kingdom; ‡ Materials Systems Engineering, Max Planck Institute for Dynamics of Complex Technical Systems, 39106 Magdeburg, Germany

**Keywords:** 2D Materials, Nanofibers, PVDF, Piezoelectric Nanogenerator, Electrospinning

## Abstract

Piezoelectric polymers have emerged as promising materials for flexible electronics, particularly in sensing and energy-harvesting applications. Their advantages over conventional inorganic piezoelectrics arise from their lower density, flexibility, ease of processing, and potentially lower costs. Many common piezoelectric polymers are bio-derived or biocompatible, making them ideal for applications in healthcare. The main disadvantage of piezoelectric polymers relative to inorganics is that their piezoelectric coefficients are usually a few orders of magnitude lower, and their low conductivity usually leads to piezoelectric nanogenerator (PENG) devices with very high output impedances, making interfacing with external circuits difficult. Among various strategies to improve the performance of piezoelectric polymers, the incorporation of two-dimensional MXenes as nanofillers has emerged as a versatile and effective approach. This Mini-Review summarizes the documented effects of MXenes on piezoelectric fluoropolymers, with a focus on electrospun nanofibers. We discuss spatially confined nanostructures and interfacial interactions that lead MXene/polymer composites to exhibit enhanced performance. We highlight some MXenes that are inherently piezoelectric, while also discussing how conventional MXenes promote piezoelectricity in polymers, in addition to improvements in other areas, such as dielectric properties, charge transport, and mechanical properties. Finally, we outline future research directions for further enhancement techniques for the piezoelectric performance of MXene-based polymer composites.

## Graphical abstract

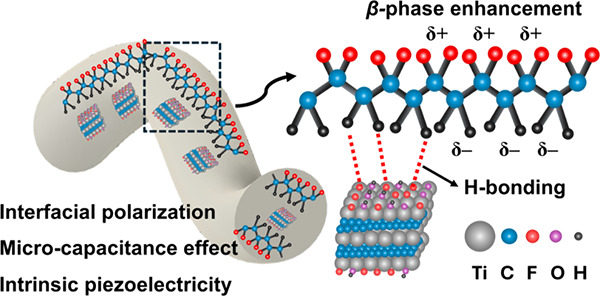

Piezoelectric materials convert mechanical deformation into electrical signals, enabling their use in sensing and energy harvesting applications. Traditional inorganic piezoelectrics such as lead zirconate titanate (PZT)^1^ exhibit high piezoelectric charge coefficients (*d*_*ij*_), but suffer from brittleness, toxicity, and poor flexibility, which limit their application in many areas. In contrast, polymer-based piezoelectrics, particularly poly­(vinylidene fluoride) (PVDF) and its copolymers, poly l-lactide (PLLA), odd polyamides (e.g., Nylon-11), and polyacrylonitrile (PAN) offer advantages including low density, high flexibility, fatigue resistance, biocompatibility, and ease of processing.^2–5^ Among them, PVDF and its copolymers such as poly­(vinylidene fluoride-*co*-trifluoroethylene) (P­(VDF-TrFE)), poly­(vinylidene fluoride-*co*-hexafluoropropylene) (P­(VDF-HFP)) and poly­(vinylidene fluoride-*co*-chlorotrifluoroethylene) (P­(VDF-CTFE)) are widely used as ferroelectric and hence, piezoelectric materials in energy-harvesting and sensing applications. PVDF exhibits several crystalline polymorphs namely α, β, γ, δ, and ε, of which the β phase is the most polar and prized for its piezoelectric and ferroelectric properties.^6,7^ However, pristine PVDF without special processing exhibits severely low β-phase content, resulting in suboptimal piezoelectric properties, which limits device performance in demanding applications.^8^ Special processing, including stretching, annealing and poling is undertaken to increase β-phase content and achieve dramatic performance improvements. Nevertheless, the intrinsic piezoelectric response of polymers remains lower than that of their piezoceramic counterparts. To address this limitation, composite approaches using nanofillers have been widely explored.^4^

MXenes are a family of transition metal carbides, nitrides, and carbonitrides, which usually occur as micro/nano-flakes, and possess unique properties including metal-like conductivity, large surface area, tunable surface chemistry, and excellent mechanical compliance. Among emerging fillers, MXenes have gained remarkable interest due to their unique structure and multifunctional properties. MXenes have a general formula M_*n*+1_X_*n*_T_*x*_, where M = transition metals, X = carbon (C) or nitrogen (N), and T = surface functional groups such as oxide (−O), hydroxyl (−OH), and fluoride (−F).^9^ By appropriately selecting M, X, and T, the electrical and mechanical properties as well as the surface chemistry of MXenes can be tailored, resulting in strong interfacial interactions with polar polymers. This makes them promising modifiers for tuning the interfacial interactions to obtain a controlled microstructure in the piezoelectric polymers. A timeline summarizing the key milestones in the development of MXenes in the piezoelectric field is shown in Figure 1, which includes theoretical predictions of intrinsic piezoelectricity, experimentally observed piezoelectric behavior, and the subsequent development of MXene/polymer piezoelectric composites and devices.

**Figure 1:**
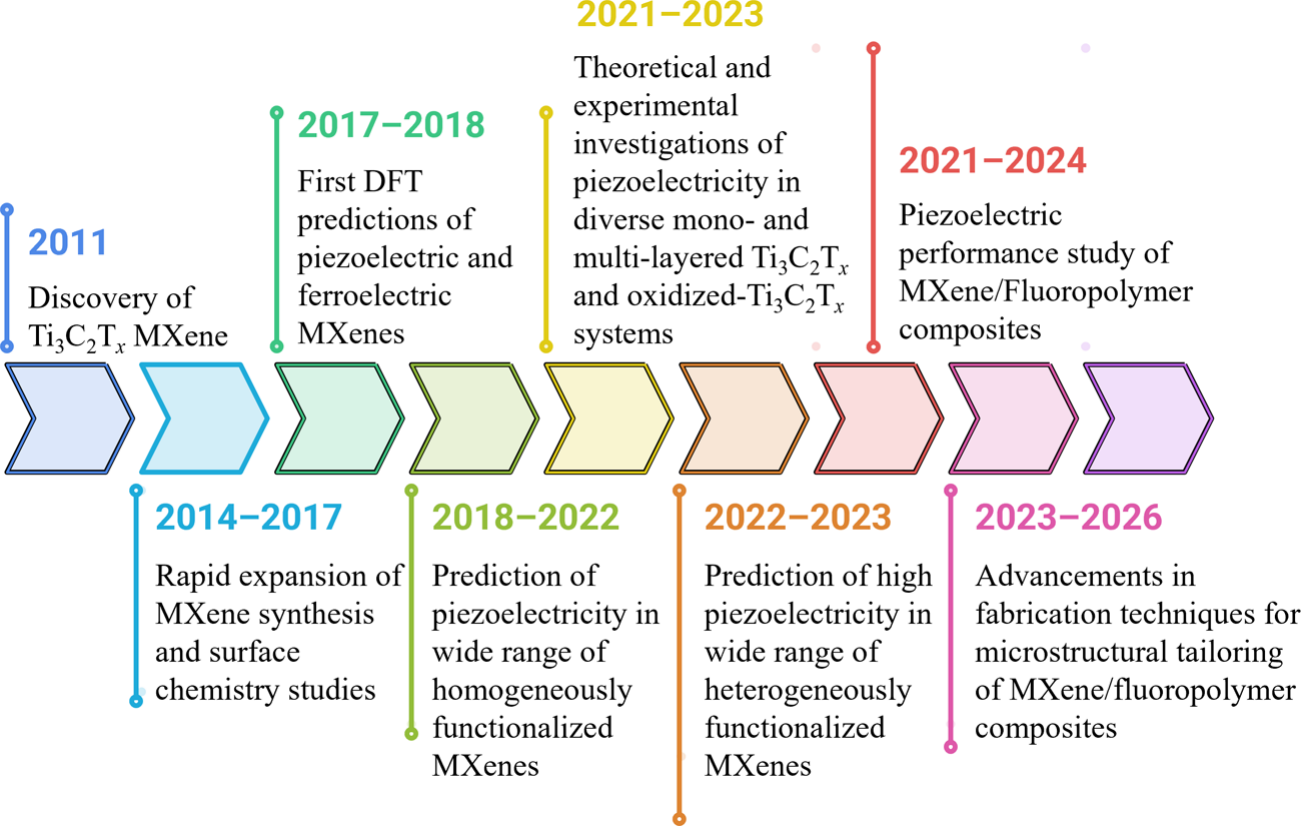
A timeline of major discoveries in piezoelectricity of MXenes and their fluoropolymer-based composites.

In this Mini-Review, we explore the intrinsic piezoelectricity of MXenes, and the effect of traditional MXenes on piezoelectric fluoropolymers. The influence of MXenes on the fluoropolymer PVDF is illustrated in Figure 2. By examining the inherent physical and chemical properties of diverse piezoelectric polymers and various MXenes, many of which are themselves piezoelectric, we arrive at an understanding of their synergy upon combination. We proceed thereafter to highlight opportunities for future improvement.

**Figure 2:**
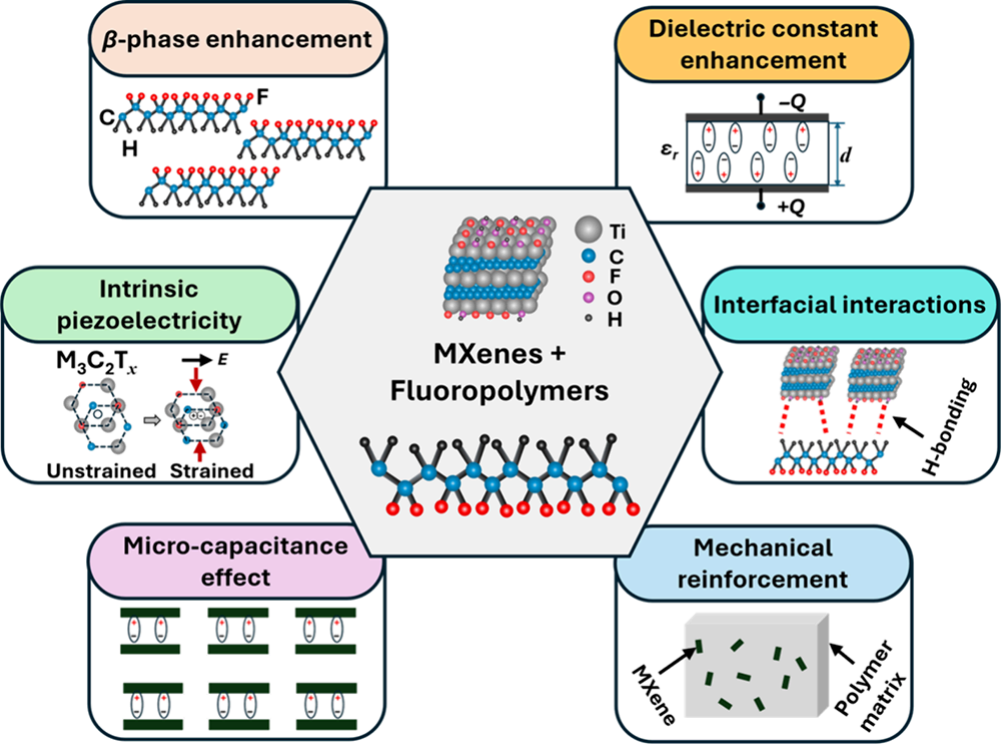
Schematic diagram of the influence of MXenes on piezoelectric fluoropolymers.

## 1 MXenes: Structure and Properties

MXenes are derived from MAX phases. MAXs are a class of layered ternary carbides and nitrides with the general formula M_*n*+1_AX_*n*_, where M is a transition metal, A is a group 13 or 14 element (e.g., Al, Si, P, S, In, Sn, etc.), X is C and/or N and *n* = 1–4. Through selective etching of the A layer from the MAX phases, MXenes (M_*n*+1_X_*n*_T_*x*_) are derived, where T = surface terminal groups originated from the etchants. Representative MXene structures and compositions are shown in Figure 3a.^10^ For example, the MXene Ti_3_C_2_T_*x*_ is derived from the MAX Ti_3_AlC_2_ phase after selective etching of Al layer. The crystal structures of Ti_3_C_2_T_*x*_ MXene and its precursor Ti_3_AlC_2_ MAX are illustrated in Figure 3b.^11^ The parent Ti_3_AlC_2_ MAX phase exhibits a hexagonal structure (space group P63/mmc), and after Al etching, the Ti_3_C_2_T_*x*_ MXene largely preserves the hexagonal framework.^11^ In this structure, Ti and C atoms are alternately arranged, and the C atoms are located at the center of each Ti octahedron. The surface terminations (T_*x*_) stabilize the material by balancing electric dipole moments and impart unique electronic properties; for instance, −OH-terminated Ti_3_C_2_T_*x*_ behaves as a narrow-bandgap semiconductor (∼0.40 eV). Structurally, a monolayer Ti_3_C_2_T_*x*_ consists of a central Ti layer sandwiched between C layers and outer Ti layers, with surface Ti atoms bonded to terminal functional groups (Figure 3b).^11^

**Figure 3:**
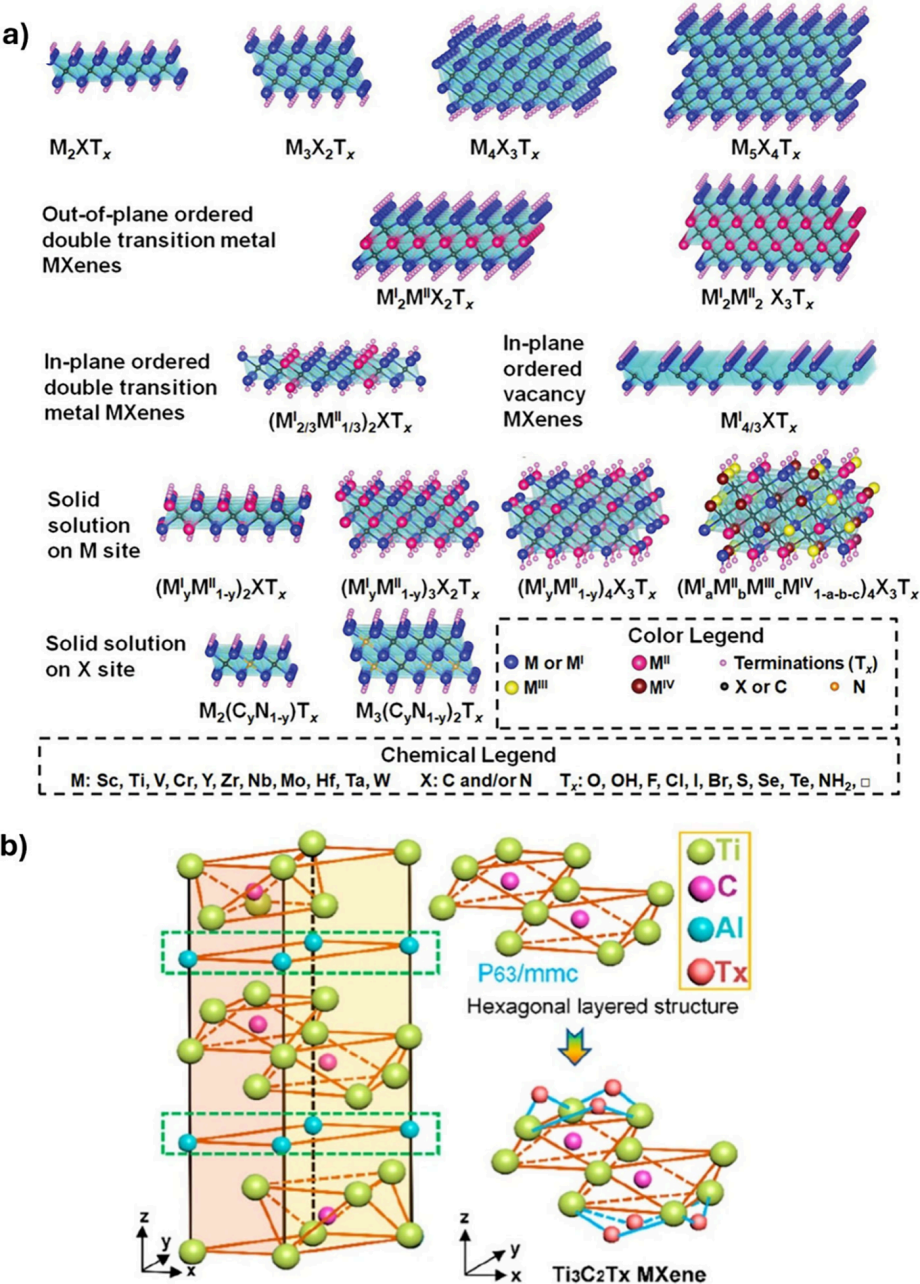
(a) Typical MXene structures and compositions. Reproduced with permission from ref 10. Copyright 2021 Wiley-VCH. (b) Crystal structure of Ti_3_AlC_2_ MAX phase (left), the hexagonal crystal structure of Ti_3_C_2_ MXene (upper right), and the crystal structure of Ti_3_C_2_T_*x*_ MXene with functional groups (lower right). Reproduced from ref 11. Copyright 2021 American Chemical Society.

MXenes possess several attributes that make them highly attractive for piezoelectric polymer composites, including high electrical conductivity (∼10,000 S cm^–1^),^12^ large specific surface area, tunable surface chemistry, strong interfacial interactions with polymers, and excellent mechanical flexibility and processability. Collectively, these features enable MXenes to function not only as conductive fillers but also as active components that regulate polymer crystallization and dipole alignment.

## 2 Piezoelectric MXenes

Achieving out-of-plane piezoelectricity in two-dimensional (2D) materials requires the formation of asymmetric polar structures. MXenes are particularly promising in this regard due to their abundant surface functional groups and compositional tunability.

### 2.1 Theoretically Predicted Piezoelectric MXenes

First, Chandrasekaran et al.^13^ employed first-principles calculations to demonstrate significant polarization in oxygen-functionalized scandium carbide MXene (Sc_2_CO_2_), revealing both in-plane and out-of-plane polarization. They reported three possible structural configurations for oxygen-functionalized MXenes: carbon-top, metal-top, and mixed, defined by the relative positions of surface oxygen atoms with respect to the bare MXene lattice. In contrast to carbon-top and metal-top configurations, the mixed configuration features oxygen atoms above carbon sites on one side and above metal sites on the opposite side. Unlike the symmetric carbon-top and metal-top structures, the mixed configuration lacks inversion symmetry and therefore can exhibit an intrinsic dipole moment. Ei-Kelany et al.^14^ also confirmed the same configurations as shown in Figure 4a. Khazaei et al.^15^ reported that single-layered (Mo_2/3_Sc_1/3_)_2_C, (Mo_2/3_Y_1/3_)_2_C, (W_2/3_Sc_1/3_)_2_C, and (W_2/3_Y_1/3_)_2_C, which are known as *i*-MXenes (*i* denotes *in-plane* chemical order), functionalized with −O–, are semiconductors with indirect band gaps and are promising for exceptional piezoelectric applications. Tan et al.^16^ further demonstrated that M_2_CO_2_ MXenes (M = Sc, Y, La) exhibit in-plane piezoelectric responses comparable to those of 2H-MoS_2_. Later, Zhang et al.^17^ presented three types of surface-functionalized ferroelectric MXene phases (type-I: Nb_2_CS_2_, Ta_2_CS_2_, Zr_2_CO_2_H_2_, and Hf_2_CO_2_H_2_; type-II: Sc_2_CO_2_ and Y_2_CO_2_; and type-III: Sc_2_CS_2_ and Y_2_CS_2_). Li et al.,^18^ Zhang et al.,^19^ and Ei-Kelany et al.^14^ separately reported the piezoelectricity of M_2_CT_2_ or M_2_CTT' MXenes, where M = transition metals (Sc, Y, Z, Hf, Ti, La), T and T' = surface functional groups (O, S, Se, Te, F, Cl), for homogeneous (T=T') and heterogeneous (T≠T') functionalization (Figure 4b).

**Figure 4:**
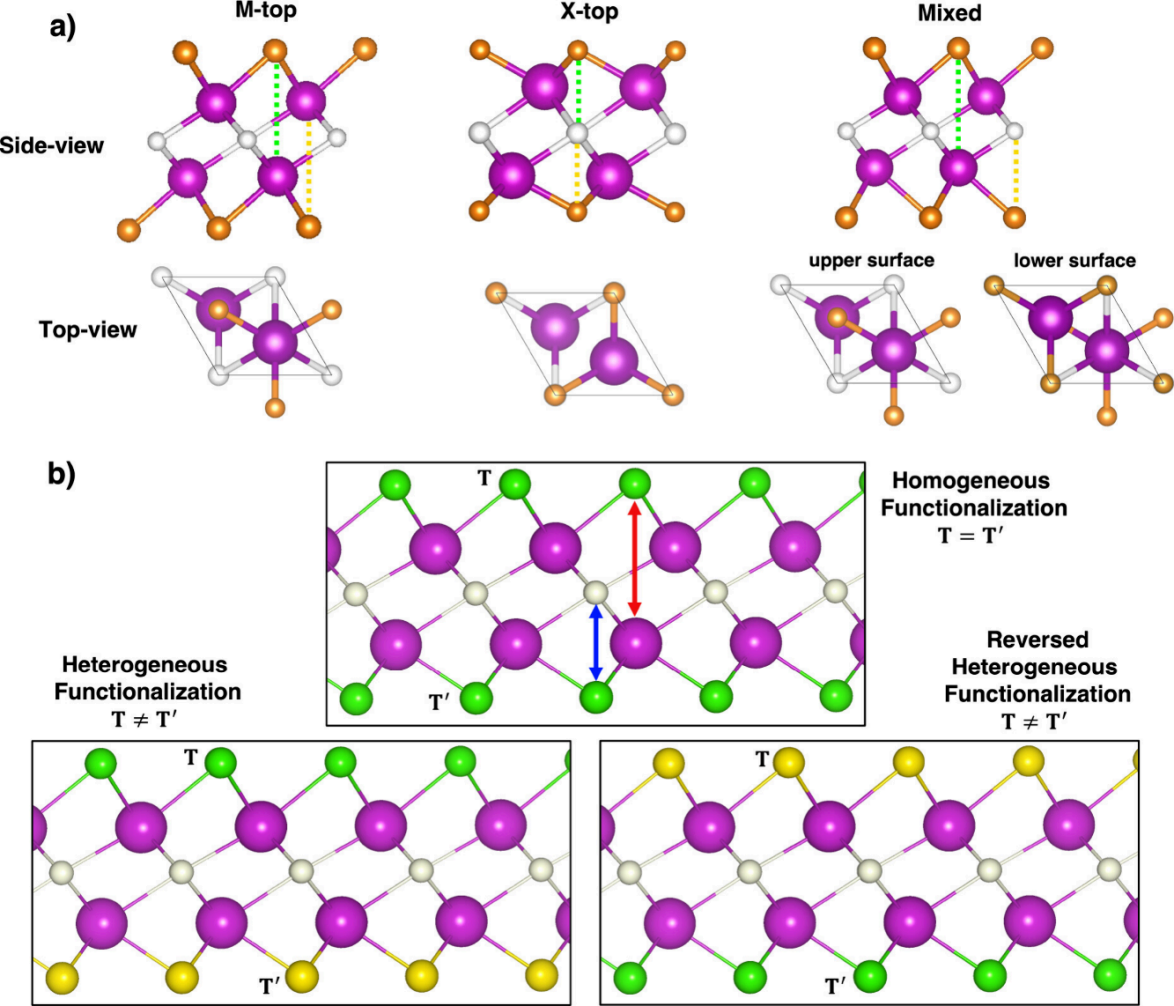
(a) Three possible structural configurations for functionalized MXenes (M_2_CT_*x*_): metal (M)-top, carbon (X)-top, and mixed configurations. (b) Various functionalization types of M_2_CTT′: homogeneous (T = T′), heterogeneous (T ≠ T′), and reversed heterogeneous (T ≠ T′); T and T′ are taken as O, S, Se, Te, F, Cl. Violet = metal, white-gray = carbon, gold = oxygen, green and yellow for functionalized atoms (T and T′). Reproduced from ref 14. Copyright 2023 American Chemical Society.

Beyond these, the intrinsic piezoelectricity of the popular Ti_3_C_2_T_*x*_ MXene in both monolayer and multilayer forms has been well studied.^11,20–22^ The crystalline structure and piezoelectric behavior of Ti_3_C_2_T_*x*_ is shown in Figure 5. Pristine Ti_3_C_2_ is centrosymmetric and exhibits no dipole moment. However, asymmetric surface functionalization induces a permanent out-of-plane dipole, indicating piezoelectric potential. Mixed terminations further enhance dipole formation regardless of symmetry configuration.^22^

**Figure 5:**
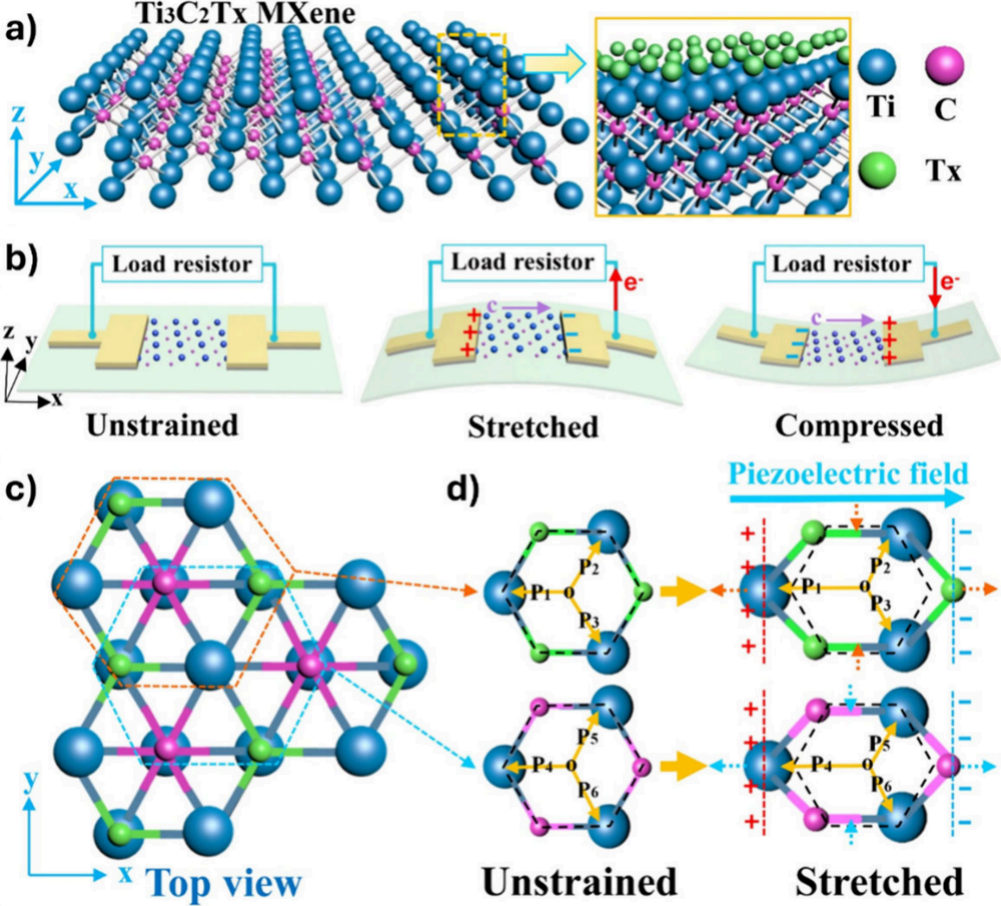
Crystal structure analysis of the Ti_3_C_2_T_*x*_ MXene. (a) Atomic structure diagrams of Ti_3_C_2_-based MXene (left) and Ti_3_C_2_T_*x*_ MXene with functional groups (right). (b) Operation scheme of the monolayer Ti_3_C_2_T_*x*_ MXene piezoelectric device, by bending the PET substrate outward and inward to realize tensile and compression strains of the MXene. (c) Top view of the crystal structure of the monolayer Ti_3_C_2_T_*x*_ MXene. (d) Tensile deformation of the simplified T_*x*_–Ti hexagonal structure (top) and Ti–C hexagonal structure (bottom), leading to the piezoelectric polarization and generating the piezoelectric electric field. Reproduced with permission from ref 20. Copyright 2021 Elsevier.

### 2.2 Experimentally Demonstrated Piezoelectric MXenes

Tan et al.^11,20^ experimentally validated the intrinsic piezoelectric response on monolayer Ti_3_C_2_T_*x*_, confirming that cyclic mechanical strain produces stable oscillating voltage and current outputs (Figure 5a–d). Jiang et al.^21^ demonstrated the potential of oxide-functionalized Ti_3_C_2_T_*x*_ MXenes for piezoelectric nanogenerators through combined experimental and theoretical investigations. Surface oxidation introduces functional groups that break lattice centrosymmetry, inducing directional piezoelectricity in the Ti_3_C_2_T_*x*_–O phase.

The development of piezoelectric MXenes from theoretical predictions to experimental observations is summarized in Table 1.

**Table 1:** Summary of the Development of Piezoelectric MXenes

MXene system	symmetry-breaking origin	method and status	ref(s)
M_2_CO_2_ (M = Sc, Y, Zr)	Intrinsic noncentrosymmetric functionalized structure, three typical configurations. C-top, M-top, and mixed configurations. In the mixed configuration, both in-plane and out-of-plane polarization were observed.	Predicted via DFT	^13^
Oxygen-terminated i-MXenes: (Mo_2/3_Sc_1/3_)_2_C, (Mo_2/3_Y_1/3_)_2_C, (W_2/3_Sc_1/3_)_2_C, (W_2/3_Y_1/3_)_2_C	Intrinsic lattice asymmetry	Predicted via DFT	^15^
M_2_CT_2_ MXenes (M = Sc, Ti, Y, Nb, Zr, La, Ta, Hf; T = O, OH, S, Se, Te, F, Cl): Sc_2_CO_2_, Sc_2_CF_2_, Sc_2_CS_2_, Sc_2_CCl_2_, Y_2_CO_2_, Y_2_CF_2_, Y_2_CS_2_, Nb_2_CS_2_, Y_2_CCl_2_, Zr_2_CO_2_, Hf_2_CO_2_, Ti_2_CO_2_, La_2_CO_2_, Hf_2_CO_2_H_2_, Zr_2_CO_2_H_2_, Ta_2_CS_2_	Intrinsic lattice asymmetry. Three types of structure were predicted based on the position of the carbon atom:	Predicted via DFT	^16–19^
	Type-I: Nb_2_CS_2_, Ta_2_CS_2_, Zr_2_CO_2_H_2_, Hf_2_CO_2_H_2_		
	Type-II: Sc_2_CO_2_, Y_2_CO_2_		
	Type-III: Sc_2_CS_2_, Y_2_CS_2_		
M_2_CTT′ (M = Sc, T and T' = (O, S, Se, Te, F, Cl), for homogeneous (T=T') and heterogeneous (T≠T') functionalization)	Intrinsic lattice asymmetry. Heterogeneous functionalization, strong dipole asymmetry between upper and lower surfaces	Predicted via DFT	^14^
Ti_3_C_2_T_*x*_ (T_*x*_ = −O, −OH, −F)	Functional-group-induced inversion symmetry breaking and dipole formation	Predicted via DFT and experimentally demonstrated via PFM and PENG	^11^,^20–24^
Oxidized Ti_3_C_2_T_*x*_–O	Oxidation-induced lattice distortion. Enhanced noncentrosymmetry and dipole polarization	Predicted via DFT and experimentally demonstrated via PFM and PENG	^21^

The piezoelectric response of Ti_3_C_2_T_*x*_ was experimentally probed using piezoresponse force microscopy (PFM). A ∼140 nm thick flake exhibited clear piezoresponse signals, including characteristic amplitude contrast and a butterfly shaped loop, confirming piezoelectric behavior. The extracted piezoelectric coefficient (*d*_33_) reached ∼192 pm V^–1^, suggesting potential for mechanical energy harvesting and piezocatalytic applications (Figure 6a,b).^22^ The *d*_33_ values reported in pm V^–1^ are generally associated with the converse piezoelectric effect and are commonly obtained by PFM, whereas values reported in pC N^–1^ typically describe the direct piezoelectric effect measured from stress-induced charge generation. Zhang et al. also examined the piezoelectric properties of Ti_3_C_2_T_*x*_ using PFM.^23^ The observed phase hysteresis and butterfly shaped amplitude loops confirm its intrinsic piezoelectric behavior (Figure 6c,d). Furthermore, Arya et al.^24^ reported a nanoscale piezoelectric response in surface-functionalized Ti_3_C_2_T_*x*_ employing PFM, and revealed an unusually high (*d*_33_) of ∼180 pm V^–1^ (Figure 6e–h).^24^ It should be noted that the large *d*_33_ (≈180–190 pm V^–1^) for Ti_3_C_2_T_*x*_ MXenes are generally obtained from local PFM measurements and represent the nanoscale electromechanical response of individual or localized MXene regions. Such values should not be directly interpreted as macroscopic piezoelectric coefficients comparable to those measured in bulk piezoelectric ceramics such as PZT (*d*_33_ ≈ 200–700 pC N^–1^).^25^ In practical devices, the effective piezoelectric response depends on factors including MXene alignment, flake stacking, surface termination distribution, interfacial coupling, charge screening, oxidation state, and composite architecture. Therefore, reported PFM-derived piezoelectric coefficients should be regarded as indicators of intrinsic piezoelectric potential rather than direct predictors of macroscopic device performance.

**Figure 6:**
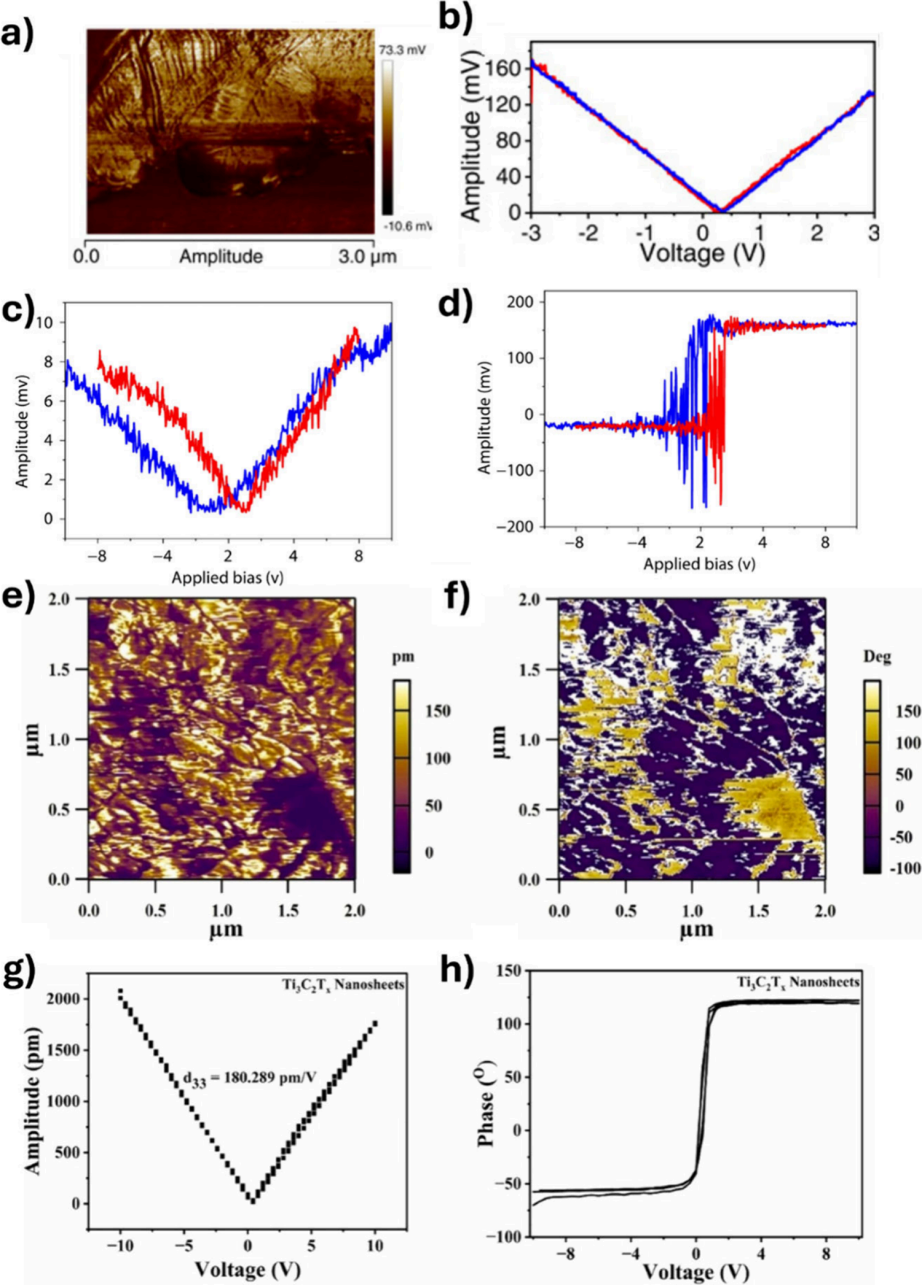
Piezoresponse force microscopy (PFM) study. (a) Amplitude image and (b) amplitude–voltage plot of Ti_3_C_2_T_*x*_. Reproduced from ref 22. CC BY 4.0. (c) Butterfly amplitude loop and (d) phase curve of Ti_3_C_2_T_*x*_. Reproduced from ref 23. CC BY 4.0. (e, f) Amplitude and phase images of the MXene Ti_3_C_2_T_*x*_ nanosheets, respectively; (g) amplitude–voltage butterfly loop and (h) phase–voltage hysteresis loop, elucidating the 180° phase change in the Ti_3_C_2_T_*x*_ nanosheets. Reproduced with permission from ref 24. Copyright 2025 Elsevier.

## 3 Piezoelectric Fluoropolymers

### Poly­(vinylidene fluoride) (PVDF)

In PVDF, the −CH_2_–CF_2_ units are arranged in different conformations (TTTT, TGTG′, and TTTGTTTG′; T = trans, G/G′ = gauche^+^/gauche^−^) and dipole packing (parallel or antiparallel), providing five possible crystalline phases: α, β, γ, δ, and ε.^6,7^ The chain conformations of these phases are shown in Figure 7. The β phase shows the highest net polarization, whereas α and ε phases show a net polarization of zero, and the γ and δ phases show a net polarization of approximately half the polarization of β. PVDF crystallizes to the most stable nonpolar α phase from the melt or solutions. The physical stretching at high temperature, or annealing or poling with a strong electric field or a combination of them, converts the α to β and other polar phases.^7^ A poled PVDF film shows *d*_33_ of 10–34 pC N^–1^.^4,26^

**Figure 7:**
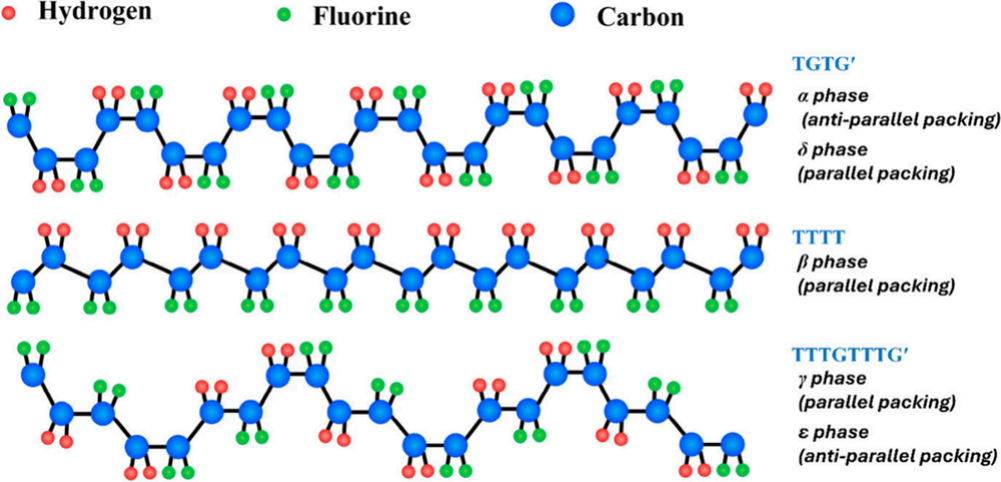
Schematic representation of the chain conformation for the α/δ, β, and γ/ε phases of poly­(vinylidene fluoride) (PVDF). Adapted from ref 27. CC BY 4.0.

### Copolymers of PVDF

P­(VDF-TrFE) is a copolymer of vinylidene fluoride (VDF) and trifluoroethylene (TrFE), containing 20–45 mol % TrFE, exists in different crystalline phases, including (α, β, γ, δ, and ε) as observed in PVDF. P­(VDF-TrFE) crystallizes from either its melt or solution predominantly in the β phase without the need for stretching, and results in stronger remanent polarization. The *d*_33_ of poled P­(VDF-TrFE) is reported to be 25–40 pC N^–1^.^4^ Similarly, the copolymer VDF and tetrafluoroethylene P­(VDF-TFE) shows higher β-phase content remanent polarization than the homopolymer PVDF. Other piezoelectric copolymers, such as P­(VDF-HFP) and P­(VDF-CTFE), show pronounced piezoelectricity from β-phase crystallization with higher remanent polarization than PVDF. The *d*_33_ of PVDF-HFP was reported to be ∼18 pC N^–1^.^28^ A terpolymer built with VDF, TrFE and CTFE, P­(VDF-TrFE-CTFE) demonstrates higher polarization than PVDF-TrFE. The *d*_33_ of P­(VDF-TrFE-CTFE) was reported to be twice that of P­(VDF-TrFE) due to saturation polarization.^29^

Operations such as drawing, annealing and poling can be used to transform these polymers into their desirable phases. These operations are often circumstantial to the fabrication method (e.g., electrospinning, solution-casting, spin-coating, hot-pressing, tape casting, etc.), and so the in-built properties of a polymer specimen are usually related to the device fabrication parameters. Electrospinning, which produces continuous polymer fibers (diameters: 10 nm to 10 μm) by stretching charged polymer jets under a strong electric field, in particular promotes molecular chain alignment and enhances the electroactive β or γ phase in ferroelectric fluoropolymers.^30^ Although in principle β-PVDF has the potential to display the highest net polarization due to its incredibly disparate electronegativities (F^δ−^ and H^δ+^) across a relatively slender chain, in practice the copolymers with a natural preference for the β-phase tend to perform better in lab-fabricated devices.

## 4 Effect of MXene Nanofillers in Piezoelectric Fluoropolymers

To further enhance the piezoelectric effect of the fluoropolymers to compete with the more established and highly responsive inorganic piezoelectrics, their electromechanical coupling can be enhanced by adding highly polar inorganic nanofillers. Incorporation of MXenes (with surface functionalization −OH, −O, and −F) in the polymer matrix enhances the electroactive β-phase and thereby increases the piezoelectric coefficient. Moreover, the inclusion of MXenes further improves the dielectric constant and mechanical strength of the polymer composites. The effect of MXene inclusions in the piezoelectric fluoropolymer matrix is discussed below.

### 4.1 β-Phase Enhancement

MXenes enhance the β-phase formation of the piezoelectric fluoropolymers, playing effective roles through (1) H-bonding, electrostatic force and interfacial polarization, (ii) electrospinning, and (iii) postpoling process. MXenes nanosheets with surface terminations −OH, −O, and −F promote H-bonding, electrostatic attraction, and electron–dipole interactions within PVDF and its copolymers. These interactions between the surface terminations and the −CH_2_/–CF_2_ dipoles induce in situ alignment of −CH_2_/–CF_2_ units into polar packing, driving a transition from randomly coiled to all-trans chain conformations.^31^ Thus, MXene nanosheets act as “β-phase nucleating sites”, forcing the polymer chains to align in the polar conformation.^32–34^ We separately discussed here the role of MXenes on H-bonding, electrospinning, and postpoling for different MXene/fluoropolymer composites.

#### 4.1.1 H-Bonding, Electrostatic Force, and Interfacial Polarization

##### MXene/PVDF

The intermolecular H-bonding formed between the surface functional groups of MXenes and the −CH_2_– group of PVDF induced the intercalation and confinement of PVDF between MXene nanosheets.^32,35,36^ Ti_3_C_2_T_*x*_ altered the dipole moment content of the PVDF polymer and provided an inducible force that caused crystalline transformation of PVDF from the α phase to the β phase.^31^ The anchoring of the polar subunits of PVDF with Ti_3_C_2_T_*x*_ via H-bonding, and the consequent change of chain confirmation, is schematically shown in Figure 8a.^31^ A phase-field simulation and molecular dynamics (MD) calculations were employed comparing −OH and =O terminated Ti_3_C_2_T*_*x*_*, to reveal that −OH terminations strengthen H-bonding interactions with the fluoropolymer matrix (Figure 8b,c). MD simulations showed that Ti_3_C_2_(OH)_2_ platelets induce a higher fraction of all-trans (TTTT) conformations in PVDF chains compared to Ti_3_C_2_O_2_. This highlights the role of H-bonding in guiding polymer chain arrangement and orientation toward the all-trans (polar β) phase, thereby enhancing spontaneous polarization and the piezoelectric performance of the fluoropolymer composites.^31^ Zu et al.^37^ elucidated that the surface functional groups of MXene stabilized molecular dipoles and showed a stronger affinity (lower binding energy) for the β phase of PVDF. Through DFT calculation, they reported that MXene/PVDF composite exhibited a significantly higher dipole moment than pure PVDF under identical external stress. Additionally, a quantitative breakdown of the binding energy indicated that the interfacial adhesion was primarily governed by electrostatic interactions and van der Waals forces (Figure 8d).^37^

**Figure 8:**
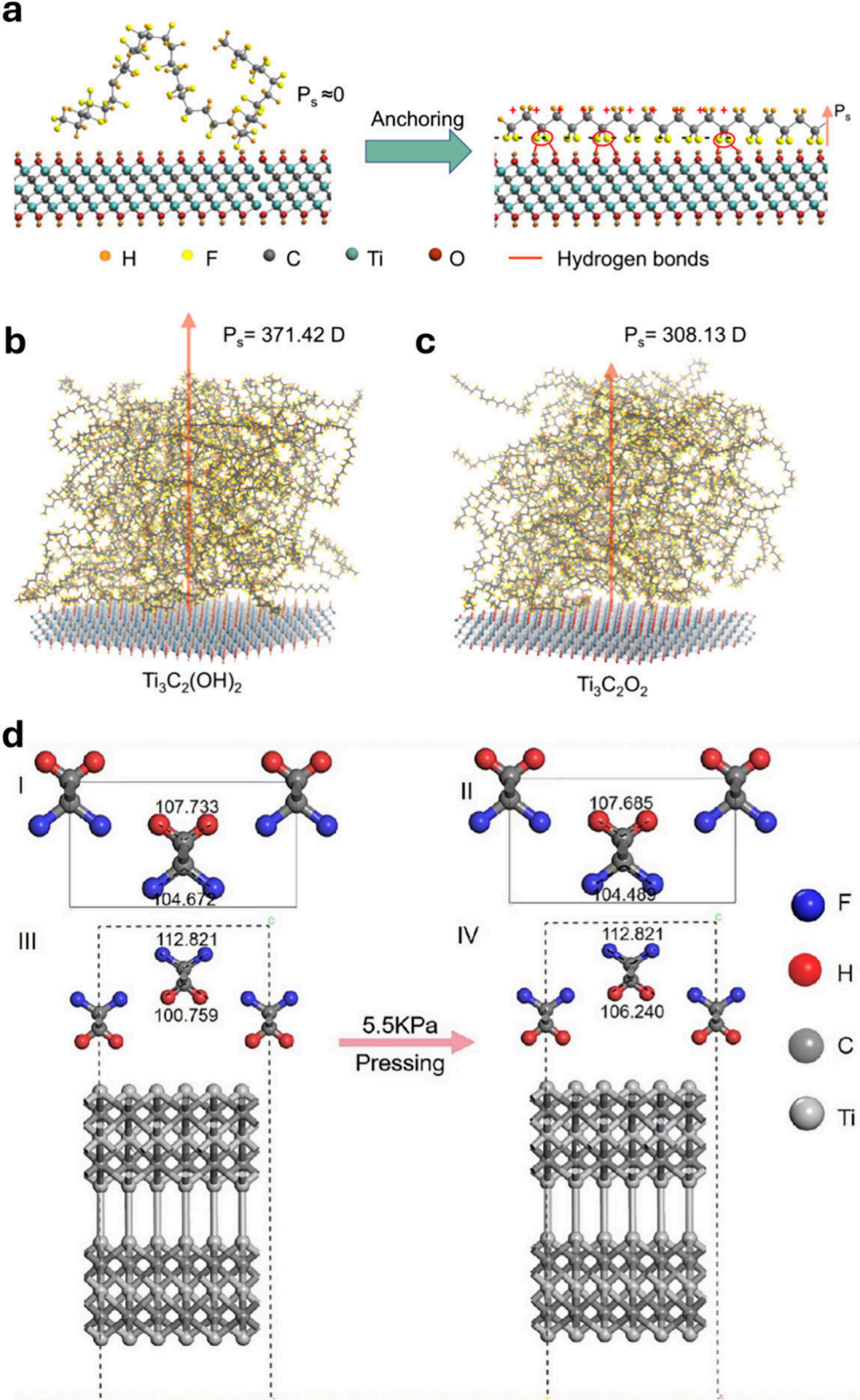
(a) Schematic of in situ stretching and alignment of PVDF polymer chain via surface terminations on Ti_3_C_2_T_*x*_ nanosheets to upgrade the spontaneous polarization (*P*_s_); (b, c) final snapshots from molecular dynamics (MD) simulations of the polarization of PVDF polymer film on the Ti_3_C_2_(OH)_2_ flakes and Ti_3_C_2_O_2_ flakes, respectively. Reproduced from ref 31. CC BY 4.0. (d) Changes in molecular structure under different conditions. Reproduced with permission from ref 37. Copyright 2025 Elsevier.

##### MXene/P­(VDF-TrFE)

The addition of MXene in P­(VDF-TrFE) resulted in similar behavior as observed in PVDF, enhancement of the β-phase content and a higher *d*_33_ due to the intermolecular H-bonding between −CH_2_ group and MXene surface functional groups.^38,39^ Shepelin et al.^40^ uncovered through MD simulations a “polarization locking” phenomenon in P­(VDF-TrFE), where the polymer’s dipole units are oriented perpendicular to the basal plane of 2D Ti_3_C_2_T_*x*_ MXene nanosheets. They show that adding the Ti_3_C_2_T_*x*_ (T_*x*_ = OH) nanosheets to P­(VDF-TrFE) in solution enables the dipoles to align perpendicular to the basal plane of the Ti_3_C_2_T_*x*_ nanosheets via electrostatic interactions at the interface, as illustrated in Figure 9. Strong interfacial electrostatic interactions induce this locked polarization, resulting in a *d*_33_ of −52.0 pC N^–1^, exceeding that of conventionally poled P­(VDF-TrFE) (∼ −38 pC N^–1^).^40^ Su et al.^31^ and later Ao et al.^41^ further confirmed −OH-terminated MXene nanosheets enhance the β-phase of P­(VDF-TrFE) via H-bonding, increasing net spontaneous polarization from 0.56 to 31.41 D.^41^

**Figure 9:**
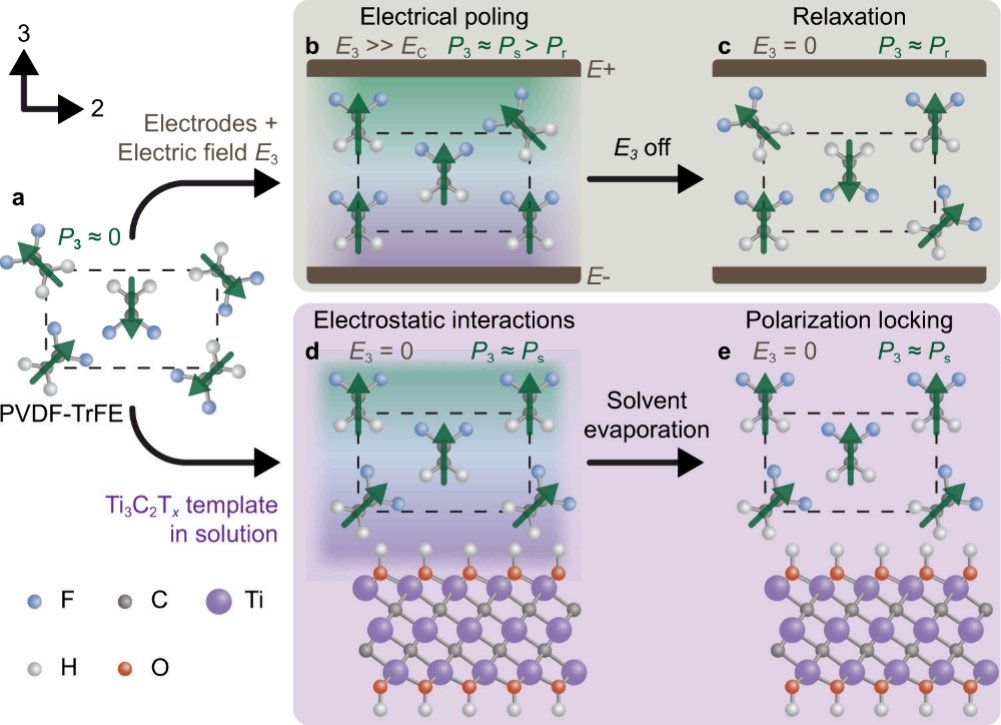
(a) As-deposited β-phase P­(VDF-TrFE) chains exhibit randomly oriented dipoles (green arrows), giving negligible net polarization (*P*). (b) During electrical poling, electrodes apply an electric field (*E* ≫ *E*_c_) perpendicular to the film, aligning dipole moments toward the spontaneous polarization (*P*_s_). (c) After removing *E*, the chains partially relax to the remnant polarization (*P*_r_). (d) Incorporating Ti_3_C_2_T_*x*_ (T_*x*_ = OH) nanosheets into the P­(VDF-TrFE) solution enables dipole alignment via interfacial electrostatic interactions without external poling. (e) Upon film deposition, Ti_3_C_2_T_*x*_ nanosheets align parallel to the substrate, and solvent evaporation locks the dipoles at *P*_s_ with minimal relaxation. Reproduced from ref 40. CC BY 4.0.

#### 4.1.2 Effect of MXenes on Electrospinning

MXenes also influence the nanofiber fabrication using the electrospinning process. MXenes reduce the viscosity of the polymer solution and simultaneously increase its conductivity, thereby enhancing the tensile forces acting on the jet during electrospinning, and producing fibers with highly stretched and well-aligned macromolecular chains. Beyond the H-bonding between PVDF and MXene nanosheets, the applied electric field induces charges on the highly conductive MXene surface, resulting in enhanced Coulombic interactions during electrospinning.^31^ The synergy between the electrospinning electric field and MXene–polymer interactions enhances polar phase content and dipole orientation along the fiber axis (Figure 10).^24,36,37,39,42^

**Figure 10:**
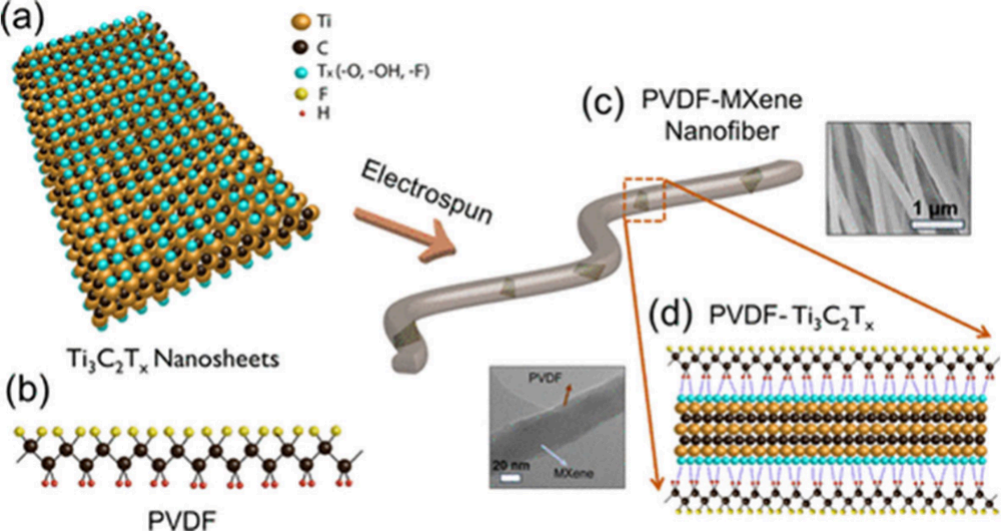
Schematic of chemical interaction between (a) the Ti_3_C_2_T_*x*_ nanosheets and (b) the PVDF molecular chain within (c) the MXene-PVDF nanofiber through (d) H-bond formation between the functional groups of Ti_3_C_2_T_*x*_ nanosheets and the −CH_2_ group from PVDF in the nanofiber. Reproduced from ref 42. Copyright 2024 American Chemical Society.

#### 4.1.3 Effect on Postpoling Treatment

MXenes not only facilitate dipole orientation during electrospinning but also aid in postpoling. Suresh et al.^43^ dealt with the orientation of polymer chains for self-poling under mild conditions. They developed anisotropic MXene/P­(VDF-TrFE) aerogels with highly aligned MXene fillers. Unidirectional freezing of the precursor solution induces alignment of MXene nanosheets and polymer chains along the crystal growth direction, driven by strong interactions between MXene surface groups (−OH/F) and P­(VDF-TrFE) chains (F–C/C–H). This process promotes the formation of abundant, preferentially oriented electroactive β phase, resulting in intrinsically aligned dipoles within the aerogel. Consequently, the piezoelectric performance of P­(VDF-TrFE) is effectively maximized without requiring additional poling.

### 4.2 Dielectric Microcapacitance Effect

It is known from the Maxwell–Wagner–Sillars (MWS) effect that the presence of conductive particles/phases in a medium (insulator) leads to dielectric loss.^44^ The sharp dielectric contrast between conductive MXene sheets and insulating polymer matrix generates interfacial polarization which increases effective permittivity and local electric field under deformation due to the MSW effect.^45^ Electrospinning can partially align conductive 2D MXene fillers along the fiber axis, creating microcapacitor-like interfacial regions within the polymer matrix as illustrated in Figure 11a,b.^46^ This microcapacitor effect is particularly important at low MXene loadings, where percolation is incomplete but interfacial polarization is maximized, enabling high piezoelectric performance without excessive conductivity that could cause leakage. Tian et al.^47^ proposed a dielectric microcapacitance strategy to enhance the piezoelectric performance of PVDF by incorporating well-aligned Ti_3_C_2_T_*x*_ MXene sheets via scalable blade coating (as illustrated in Figure 11d,e). The aligned MXene/PVDF composite achieved a significantly improved piezoelectric coefficient (∼63 pC N^–1^), attributed to enhanced permittivity and interfacial polarization via microcapacitance engineering.^47^

**Figure 11:**
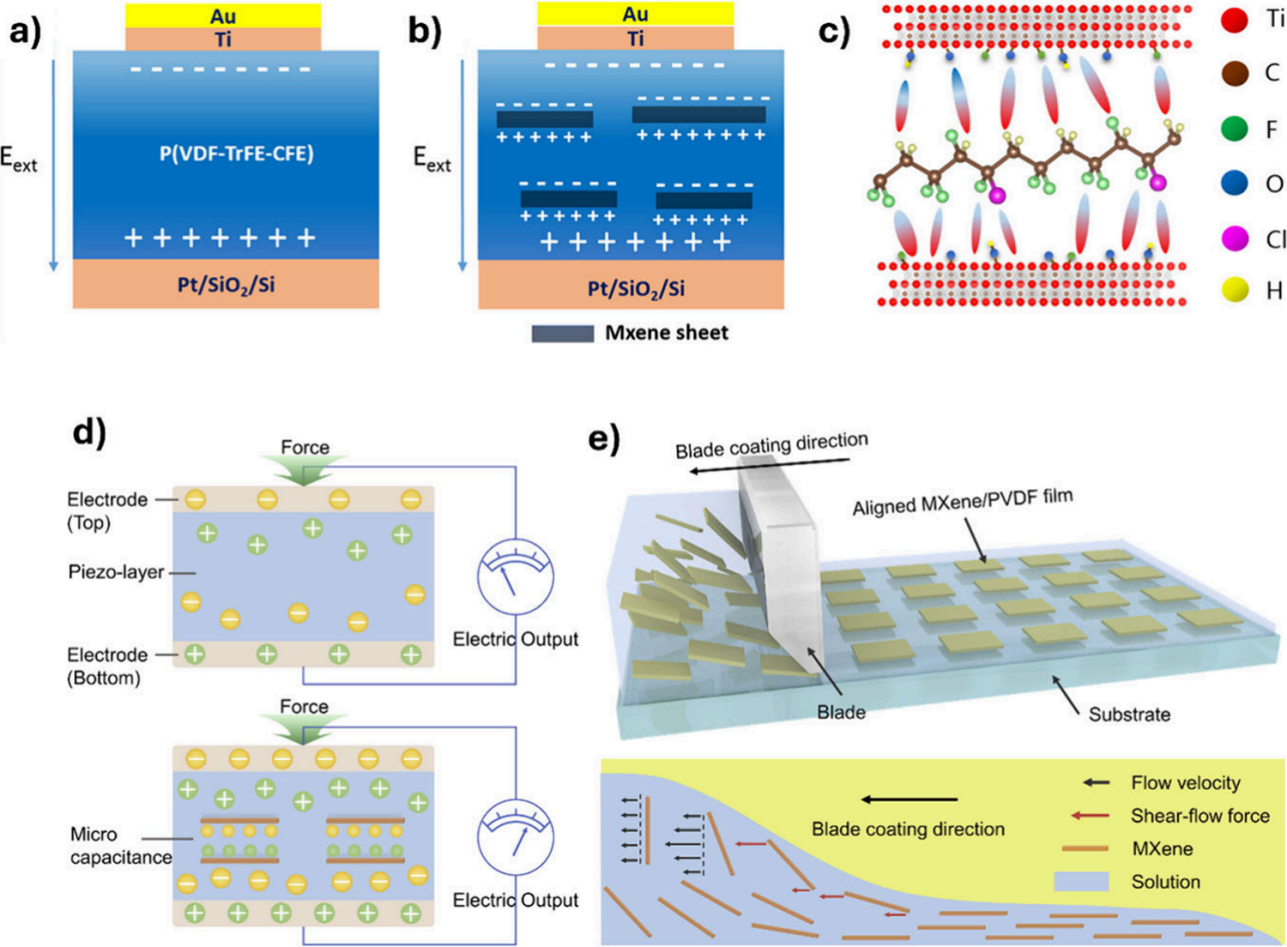
(a, b) Schematics showing polarization charge distribution in pristine P­(VDF-TrFE-CFE) and MXene/P­(VDF-TrFE-CFE) MIM capacitors under an external electric field (*E*_ext_) and (c) illustration of possible dipole formation between MXene surface terminations (F, O) and hydrogen atoms on the polymer backbone when polymer chains intercalate between MXene layers. Reproduced from ref 46. Copyright 2018 American Chemical Society. (d) Schematic illustration of dielectric microcapacitance-enhanced piezoelectricity and (e) blade-coating process (top) and theoretical schematic (bottom) of the shear-flow force-induced alignment of MXene sheets. Reproduced from ref 47. CC BY-NC-ND 4.0.

### 4.3 Effect of the Conductivity of MXenes on Dielectric Properties of Polymers

MXenes possess high electrical conductivity; therefore, their incorporation into polymer matrices significantly influences the dielectric constant, dielectric loss, and leakage current. Thus, optimization of MXene content is essential to achieve optimal loading and maintain balanced electrical performance. Tu et al.^46^ showed that in 10 wt % MXene loading in P­(VDF-TrFE-CFE) (CFE = chlorofluoroethylene), yields an ∼25-fold increase in dielectric constant with only ∼6-fold increase in dielectric loss (from 0.06 to 0.35), where the percolation threshold of dielectric constant was at 15 wt % MXene loading (as shown in Figure 12a).^46^ Dielectric constant enhancement was largely due to the charge accumulation caused by the formation of microscopic dipoles at the surfaces between the MXene sheets and the polymer matrix under an externally applied electric field. They compared the enhancement of dielectric constant with the addition of 4 wt % of MXene among P­(VDF-TrFE-CFE), P­(VDF-TrFE-CTFE), P­(VDF-TrFE), and polyvinylpyrrolidone (PVP). P­(VDF-TrFE-CFE) showed a 6-fold increment of dielectric constant (Figure 12b). Notably, with the increment of MXene loading, the conductive particles induce leakage current. Thus, an optimum loading of MXene is required to obtain the highest piezoelectric output.

**Figure 12:**
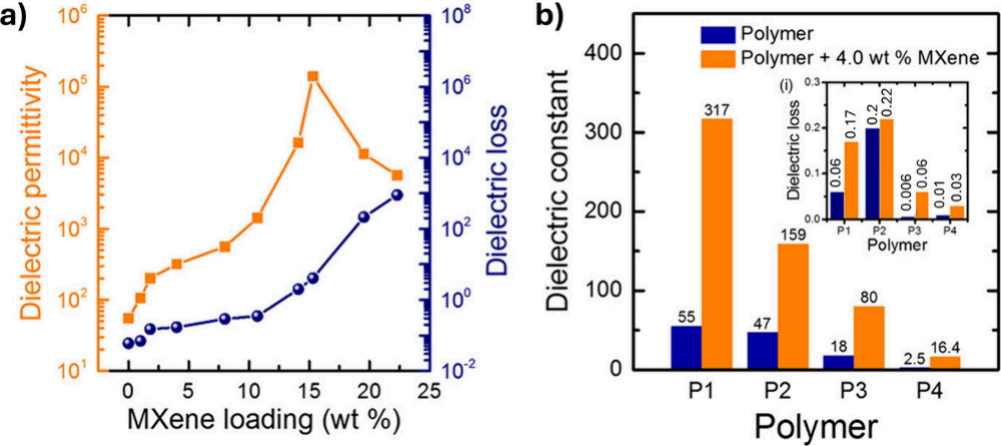
(a) Permittivity and dielectric loss of MXene/P­(VDF-TrFE-CFE) composites as a function of MXene wt % at 1 kHz. (b) Dielectric constants of various polymers with and without 4.0 wt % MXene. Inset (i): Dielectric loss of polymers containing 4.0 wt % MXene. P1, P­(VDF-TrFE-CFE); P2, P­(VDF-TrFE-CTFE); P3, P­(VDF-TrFE); P4, PVP. Reproduced from ref 46. Copyright 2018 American Chemical Society.

In several other studies, MXene has been incorporated into PVDF and/or its copolymers to enhance dielectric permittivity, suppress dielectric loss, alongside promote β-phase formation through interfacial interactions.^34,40,41,48–51^ Nayak et al.^52^ claimed that the incorporation of conductive Ti_3_C_2_T_*x*_ into PVDF markedly enhances its dielectric, piezoelectric, and ferroelectric performance. The dielectric constant increases with MXene loading up to an optimal content (∼4 wt %), corresponding to the percolation threshold, beyond which conductivity rises and dielectric behavior declines. At this composition, the composite exhibits improved impedance characteristics, reduced leakage current density, and enhanced ferroelectric behavior compared to pristine PVDF.^52^ Liu et al.^53^ carried out the DFT studies of Ti_3_C_2_T_*x*_/PVDF interfaces to reveal that dielectric enhancement is governed by H-bond-induced dipole modulation. In particular, −OH-terminated MXenes drive PVDF dipole reorientation, accompanied by interfacial charge transfer and strong electrostatic coupling. This interfacial engineering simultaneously increases dielectric permittivity and breakdown strength while suppressing dielectric loss, highlighting H-bonding as a key strategy for dipole regulation and performance optimization in MXene/PVDF dielectric composites.^53^

The dielectric permittivity enhancement achieved by MXene incorporation is often accompanied by a fundamental trade-off. As the MXene concentration approaches or exceeds the percolation threshold, the formation of conductive pathways can significantly increase dielectric loss, leakage current and reduce dielectric breakdown strength (DBS). Such effects are particularly detrimental for piezoelectric polymers that require high electric fields during poling to achieve optimal dipole alignment. A lower DBS restricts the maximum applicable poling field, reducing poling efficiency and limiting the attainable piezoelectric response. Moreover, increased leakage currents can accelerate depolarization, electrical aging, and long-term performance degradation under cyclic operation. Therefore, optimizing MXene loading requires balancing dielectric permittivity enhancement with leakage suppression to achieve superior piezoelectric performance.

It should be noted that the “optimum MXene loading” reported in the literature may depend on the performance metric considered, corresponding to the composition with the highest *d*_33_ or highest β phase content. While increasing MXene content often enhances β phase formation, interfacial polarization, and *d*_33_, it also increases dielectric permittivity and dielectric loss. The generated electric field and voltage are governed by the piezoelectric voltage coefficient (*g*_*ij*_), as defined in eq 1:

1 $$g_{ij}=\frac{d_{ij}}{\varepsilon_{0}\varepsilon_{r}}$$

where ε_r_ is the relative dielectric permittivity and ε_0_ is the vacuum permittivity.

Since the piezoelectric voltage coefficient is inversely proportional to the dielectric permittivity, compositions exhibiting the highest *d*_*ij*_ do not necessarily provide the highest voltage output or energy-harvesting efficiency. Therefore, evaluation is based on the piezoelectric figure of merit (FoM) (eq 2), which provides a more comprehensive assessment of the suitability of MXene/polymer composites for energy-harvesting applications:

2 $$\mathrm{FoM}=d_{ij}\times g_{ij}$$

For MXene/fluoropolymer systems, the 3–3 direction is the most relevant for characterization; therefore, the FoM can be calculated using eq 3 as follows::

3 $$\mathrm{FoM}=d_{33}\times g_{33}=\frac{{d_{33}}^{2}}{\varepsilon_{0}\varepsilon_{r}}$$

which reflects the balance between charge generation and dielectric screening.

### 4.4 Effect of Intrinsic Piezoelectricity of MXenes on Polymer Composites

Certain low-symmetry or functionalized MXenes have been found to be intrinsically piezoelectric.^18,20,21^ When such “piezo-MXenes” or oxidized MXene layers are coupled with piezoelectric polymers, the composite can exhibit synergistic piezoelectric response, both phases generate polarization under strain, and interfacial band alignment enhances charge separation, raising the overall effective piezoelectric coefficient. To elucidate the role of intrinsic piezoelectricity of MXenes in polymer composites, nonfluoropolymer-based composites serve as ideal examples. Arya et al.^24^ demonstrated a multilayer Ti_3_C_2_T_*x*_ MXene-based flexible PENG using surface-functionalized nanosheets embedded in polydimethylsiloxane (PDMS). The optimized composite (15 vol % MXene) produced a high output voltage of ∼76 V under vertical pressure. PFM measurement revealed a high *d*_33_ ≈ 180 pm V^–1^, attributed to symmetry breaking induced by surface terminations. These results highlight functionalized MXene as a promising active material for flexible self-powered piezoelectric devices.^24^

### 4.5 Mechanical Reinforcement, Structure Control, and Stress Transfer

MXene nanosheets act as load-bearing fillers. Their 2D geometry and high aspect ratio of enable effective stress transfer from the polymer matrix to the filler, leading to enhanced electromechanical coupling. MXene nanosheets reinforce electrospun fibrous membranes by forming multiple H-bonds and mechanical interlocks with polymer chains, leading to increased tensile strength and strain at break in optimized compositions.^41^ Enhanced mechanical robustness enables repeated bending or stretching without structural failure, which is essential for wearable and implantable devices exposed long-term cyclic loading. However, excessive MXene loading may cause fiber coarsening, surface roughness, or agglomeration, which can negatively affect mechanical homogeneity and biological responses; therefore, an optimal MXene content must balance mechanical reinforcement against structural integrity and processability.^33,52,54^

The effects of MXenes on the piezoelectric performance of fluoropolymers are summarized in Table 2. Beyond β-phase induction, the spatial orientation of MXene nanosheets plays a critical role in determining piezoelectric performance. Randomly dispersed MXene primarily acts as a nucleating and interfacial polarization agent, whereas aligned MXene structures can additionally enhance dipole orientation, anisotropic stress transfer, and polarization continuity. Recent studies employing blade coating, solvent-evaporation-assisted printing, and oriented porous architectures have demonstrated substantially higher piezoelectric coefficients and output signals compared with randomly dispersed systems. For example, aligned MXene/PVDF composites exhibited *d*_33_ values up to –63.3 pC N^–1^,^47^ while polarization-locking strategies based on aligned Ti_3_C_2_T_*x*_ nanosheets achieved *d*_33_ values of ∼–52 pC N^–1^.^40^ These results highlight that filler orientation is an important design parameter besides filler concentration and β-phase content, supporting future efforts toward controlled anisotropic MXene assembly in piezoelectric polymer nanofibers.

**Table 2:** Effect of MXenes on Different Fluoropolymers toward Piezoelectric Performance

material	fabrication method and morphology	MXene loading	MXene alignment	*d*_33_ of pristine polymer [pC N^–1^]	*d*_33_ of MXene/Polymer composite [pC N^–1^]	enhancement of piezoelectric properties	enhancement of device performance	ref
Ti_3_C_2_T_*x*_/PVDF	Blade coating, film	15 % wt ratio	Highly aligned	–24	–63.3	*d*_33_ is ∼2.64 times higher than that of pristine PVDF		^47^
Ti_3_C_2_T_*x*_/PVDF	Electrospinning, nanofiber mat	0.8 wt %	Spatially confined alignment				Output voltage is 3.97 times higher and current is 10.1 times higher than those of pristine PVDF	^35^
Ti_3_C_2_T_*x*_/PVDF	Electrospinning, nanofiber mat	6 wt %	Spatially confined alignment	3.6	11.9	*d*_33_ is ∼3.3 times higher than that of pristine PVDF	Output voltage is 7 times higher and current is 3.8 times higher than those of pristine PVDF	^37^
Ti_3_C_2_T_*x*_/PVDF	Electrospinning, nanofiber mat	Derived MXene from 2 g of MAX in 3 g of PVDF	Spatially confined alignment		61.7	*d*_33_ of aligned PVDF/Ti_3_C_2_T_*x*_ nanofibers mat is ∼4 times higher than that for random alignment	The PVDF/Ti_3_C_2_T_*x*_ composite shows an output current of 230 nA	^36^
Ti_3_C_2_T_*x*_/P(VDF-TrFE)	Solvent-evaporation assisted extrusion 3D Printing, film	0.5 wt %	Highly aligned	–28.6	–52.0	*d*_33_ is ∼1.8 times higher than that of pristine PVDF-TrFE		^40^
Ti_3_C_2_T_*x*_/P(VDF-TrFE)	A combination of blade coating and nonsolvent induced phase separation, film	2.5 wt %	Highly aligned				Output current is ∼3 times higher than that of the pristine P(VDF-TrFE) at 200 kPa	^41^
Ti_3_C_2_T_*x*_/P(VDF-TrFE)	Electrospinning followed by MXene coating, mat	6 wt %	Aligned				Output voltage is 20 times higher than that of pristine P(VDF–TrFE)	^55^

## 5 Challenges and Limitations

Despite promising piezoelectric enhancement capabilities of MXenes, the long-term stability of MXene/polymer composites remains an important challenge. Ti_3_C_2_T_*x*_ is susceptible to oxidation in the presence of moisture, oxygen, and elevated temperatures, leading to the gradual formation of TiO_2_ nanoparticles and degradation of the layered structure.^56^ Such oxidation can alter surface terminations, reduce electrical conductivity, weaken interfacial polarization, and diminish H-bonding interactions with fluoropolymer chains. Consequently, the β-phase stabilization, dielectric enhancement, and piezoelectric response reported for MXene/polymer composites may deteriorate during long-term operation. Furthermore, oxidation-induced defects may adversely affect DBS and electrical reliability. Therefore, systematic investigations of thermal aging, environmental stability, and cyclic durability are required to establish realistic performance limits for MXene-based piezoelectric devices.

## 6 Outlook

Future control of piezoelectricity in MXene–polymer composites can focus on the following complementary strategies:

### 6.1 Anisotropic Flake Alignment

The controlled anisotropic alignment of MXene nanosheets within fluoropolymer matrices represents an important yet underexplored avenue for improving piezoelectric performance. While a few studies have reported aligned MXene/PVDF architectures,^43,57^ a comprehensive understanding of the relationship between MXene orientation, dipole alignment, and electromechanical coupling is still lacking. Future efforts should therefore emphasize advanced processing strategies for directional MXene assembly, enabling optimized charge polarization, enhanced stress transfer, and superior energy-conversion efficiency in next-generation piezoelectric composites.

### 6.2 Surface Termination Engineering

Termination control offers another route to tune piezoelectric performance. MXene surface groups (e.g., −O, −OH, −F, −Cl) generate local dipoles and influence interactions with polymer matrices. Adjusting termination type and ratio can modify dipole strength, enhance nucleation of polar polymer phases, and stabilize aligned dipoles at the interface. Mixed or asymmetric terminations further break centrosymmetry and create built-in interfacial polarization.

### 6.3 Interface Engineering

In tandem with MXene surface engineering, modification of polymers must be explored to achieve most successful interfacial engineering through functionalization, hydrogen-bond design, molecular dipole templating, and insulating interfacial coatings on fluoropolymers. Such approaches could enhance β-phase stabilization, interfacial polarization, and charge transfer while suppressing leakage current and preserving DBS. Furthermore, understanding the relationship between MXene oxidation, surface chemistry evolution, and piezoelectric performance remains an important research direction.

### 6.4 Exploring Synergies between Intrinsically Piezoelectric MXenes and Piezoelectric Polymers

So far, the studies have been limited mostly to Ti_3_C_2_T_*x*_ MXenes. Thus, it is time to explore the effect of other piezoelectric MXenes such as M_2_CT_2_ or M_2_CTT′, where M = transition metals (Sc, Y, Z, Hf, Ti, La), T and T' = (O, S, Se, Te, F, Cl), (T=T' and T≠T'), on piezoelectric fluoropolymers and to explore the synergistic piezoelectric effect of MXene and fluoropolymers.

### 6.5 Scalable Fabrication

Despite significant advances at the laboratory scale, the development of scalable and reproducible manufacturing routes remains a key challenge for MXene-based piezoelectric composites. Future efforts should focus on industrially compatible processes such as roll-to-roll electrospinning, blade coating, slot-die coating, melt processing, and additive manufacturing. In particular, scalable approaches capable of simultaneously controlling MXene dispersion, orientation, and polymer crystallization will be critical for translating laboratory-scale performance into large-area flexible devices.

Although remarkable progress has been achieved in enhancing piezoelectric performance through MXene incorporation, future advances will likely depend less on increasing filler content and more on precise control of multiscale structure–property relationships. Together, controlled alignment and tailored terminations provide a framework for directional piezoelectric design, enabling MXene–polymer nanofibers with optimized anisotropy and interfacial polarization to enhance the piezoelectric properties of the composites. In addition, aligning the inherent piezoelectric MXenes to the piezoelectric polymers will synergistically enhance their piezoelectric properties for application in flexible sensors and nanogenerators.
